# Vaccination coverage and delay in vaccination of infants born in 2017 and 2018 in municipalities in the Southern region of Brazil: National Vaccination Coverage Survey 2020

**DOI:** 10.1590/S2237-96222024v33e20231206.especial2.en

**Published:** 2024-10-21

**Authors:** Karin Regina Luhm, Antonio Fernando Boing, Sotero Serrate Mengue, Neiva de Souza Daniel, Tainá Ribas Mélo, Raquel Jaqueline Farion, Ana Paula França, José Cássio de Moraes, ICV 2020 Group, Adriana Ilha da Silva, Alberto Novaes Ramos, Ana Paula França, Andrea de Nazaré Marvão Oliveira, Antonio Fernando Boing, Carla Magda Allan Santos Domingues, Consuelo Silva de Oliveira, Ethel Leonor Noia Maciel, Ione Aquemi Guibu, Isabelle Ribeiro Barbosa Mirabal, Jaqueline Caracas Barbosa, Jaqueline Costa Lima, José Cássio de Moraes, Karin Regina Luhm, Karlla Antonieta Amorim Caetano, Luisa Helena de Oliveira Lima, Maria Bernadete de Cerqueira Antunes, Maria da Gloria Teixeira, Maria Denise de Castro Teixeira, Maria Fernanda de Sousa Oliveira Borges, Rejane Christine de Sousa Queiroz, Ricardo Queiroz Gurgel, Rita Barradas Barata, Roberta Nogueira Calandrini de Azevedo, Sandra Maria do Valle Leone de Oliveira, Sheila Araújo Teles, Silvana Granado Nogueira da Gama, Sotero Serrate Mengue, Taynãna César Simões, Valdir Nascimento, Wildo Navegantes de Araújo

**Affiliations:** 1 Universidade Federal do Paraná, Departamento de Saúde Coletiva, Curitiba, PR, Brazil; 2 Universidade Federal de Santa Catarina, Departamento de Saúde Pública, Florianópolis, SC, Brazil; 3 Universidade Federal do Rio Grande do Sul, Programa de Pós-Graduação em Epidemiologia, Porto Alegre, RS, Brazil; 4 Universidade Federal do Paraná, Programa de Pós-Graduação em Saúde Coletiva, Curitiba, PR, Brazil; 5 Universidade Federal do Paraná, Pós-Graduação e Graduação em Saúde Coletiva, Curitiba, PR, Brazil; 6 Pontifícia Universidade Católica do Paraná, Escola de Medicina e Ciências da Vida, Curitiba, PR, Brazil; 7 Faculdade de Ciências Médicas da Santa Casa de São Paulo, Departamento de Saúde Coletiva, São Paulo, SP, Brazil; Universidade Federal do Espírito Santo, Vitória, ES, Brazil; Universidade Federal do Ceará, Departamento de Saúde Comunitária, Fortaleza, CE, Brazil; Faculdade Ciências Médicas Santa Casa de São Paulo, São Paulo, SP, Brazil; Secretaria de Estado da Saúde do Amapá, Macapá, AP, Brazil; Universidade Federal de Santa Catarina, SC, Brazil; Organização Pan-Americana da Saúde, Brasília, DF, Brazil; Instituto Evandro Chagas, Belém, PA, Brazil; Faculdade de Ciências Médicas Santa Casa de São Paulo, Departamento de Saúde Coletiva, São Paulo, SP, Brazil; Universidade Federal do Rio Grande do Norte, Natal, RN, Brazil; Universidade Federal do Ceará, Departamento de Saúde Comunitária, Fortaleza, CE, Brazil; Universidade Federal de Mato Grosso, Cuiabá, MT, Brazil; Universidade Federal do Paraná, Curitiba, PR, Brazil; Universidade Federal de Goiás, Goiânia, GO, Brazil; Universidade Federal do Piauí, Teresina, PI, Brazil; Universidade de Pernambuco, Faculdade de Ciências Médicas, Pernambuco, PE, Brazil; Instituto de Saúde Coletiva, Universidade Federal da Bahia, Salvador, BA, Brazil; Secretaria de Estado da Saúde de Alagoas, Maceió, AL, Brazil; Universidade Federal do Acre, Rio Branco, AC, Brazil; Universidade Federal do Maranhão, Departamento de Saúde Pública, São Luís, MA, Brazil; Universidade Federal de Sergipe, Aracaju, SE, Brazil; Secretaria Municipal de Saúde, Boa Vista, RR, Brazil; Fundação Oswaldo Cruz, Mato Grosso do Sul, Campo Grande, MS, Brazil; Fundação Oswaldo Cruz, Escola Nacional de Saúde Pública Sergio Arouca, Rio de Janeiro, RJ, Brazil; Universidade Federal do Rio Grande do Sul, Porto Alegre, RS, Brazil; Fundação Oswaldo Cruz, Instituto de Pesquisa René Rachou, Belo Horizonte, MG, Brazil; Secretaria de Desenvolvimento Ambiental de Rondônia, Porto Velho, RO, Brazil; Universidade de Brasília, Brasília, DF, Brazil

**Keywords:** Cobertura vacunal, Vacunas, Encuestas Epidemiológicas, Incertidumbre ante las vacunas, Retraso en la Vacunación, Vaccination Coverage, Vaccines, Health Surveys, Vaccination Hesitancy, Vaccination Delay

## Abstract

**Objective:**

To evaluate vaccination coverage and delay in vaccine dose administration in infants in six municipalities in the Southern region of Brazil.

**Methodology:**

National Vaccination Coverage Survey 2020, with infants born alive in 2017 and 2018, carried out from September 2020 to March 2022. Coverage of doses administered, doses administered on time and delay in dose administration were evaluated.

**Results:**

For 4681 infants analyzed, coverage for vaccines recommended up to 24 months was 68.0% (95%CI 63.9;71.8%) for doses administered and 3.9% (95%CI 2.7%;5.7%) for doses administered on time. Delay time for the majority of late vaccinations was ≤ 3 months. For some boosters, 25% of vaccine administration was delayed by ≥ 6 months.

**Conclusion:**

In addition to tracking vaccine defaulters, strategies are needed to encourage compliance with the vaccination schedule at the recommended ages.

## Study contributions

**Main results:**

Vaccination coverage for the set of vaccines recommended up to 24 months was 68.0% and 3.9% for on-time doses. Delay time for some doses exceeded six months in up to 25% of infants with delayed vaccination.

**Implications for services:**

Monitoring vaccine administration at the recommended ages is necessary, with the adoption of strategies that reinforce routine vaccination to prevent vaccination delays and abandonment.

**Perspectives:**

Primary care in surveillance and care for infants needs to reinforce actions to ensure timely vaccination. Studies to deepen knowledge of vaccination delay, determinants and strategies for their reduction are necessary.

## RESUMEN

**Objetivo:**

Evaluar la cobertura de vacunación y los retrasos en la dosis de vacuna entre lactantes en seis municipios de la región Sur de Brasil.

**Metodología:**

Encuesta Nacional de Cobertura de Vacunación 2020, con infantes nacidos vivos en 2017 y 2018, llevada a cabo de septiembre de 2020 a marzo de 2022. Se evaluó la cobertura de dosis aplicadas, dosis actualizadas y el retraso en la aplicación.

**Resultados:**

De los 4.681 lactantes evaluados, la cobertura de vacunas recomendadas hasta los 24 meses fue del 68,0% (IC 95% 63,9;71,8%) para las dosis administradas y del 3,9% (IC 95% 2,7%;5,7%) para las dosis diarias. La mayoría de las solicitudes tardías fueron ≤ 3 meses. Para algunos refuerzos, el 25% de las solicitudes se retrasaron ≥ 6 meses.

**Conclusión:**

Además de buscar morosos en la vacunación, se necesitan estrategias para incentivar el cumplimiento del calendario de vacunación en las edades recomendadas.

## INTRODUCTION

As an important public health action for disease prevention, vaccination has significantly reduced morbidity and mortality from vaccine-preventable diseases in Brazil. Driven by the National Immunization Program (*Programa Nacional de* *Imunização* - PNI), vaccination is a strategic action of the Brazilian National Health System (*Sistema Único de Saúde* - SUS) throughout the country. Vaccination is offered universally and free of charge, according to a routine vaccination schedule for the entire life cycle of the population.^1^

The success of the PNI’s actions is due to its ability to reach a large part of the population (all territories and social classes), its high rate of vaccination coverage ‒ ensuring the restriction of the spread of agents that cause vaccine-preventable diseases ‒, the quality of immunobiological products administered and administration procedures, health service accessibility and addessing existing socioeconomic and cultural barriers.^1^

Despite the relevance and positive impact of the PNI over five decades, there was a sharp drop in Brazil in the coverage of several immunobiological products between 2016 and 2021. In 2021, 14 vaccines were available on the routine vaccination schedule for children under 2 years of age,^2^ however Brazil did not achieve 75% coverage for any immunobiological product that year. Between 2016 and 2021, coverage of pneumococcal vaccine fluctuated between 95.0% to 74.8%, meningococcal vaccine coverage between 91.7% and 72.2%, and Bacillus Calmette-Guérin (BCG) vaccine coverage between 95.6% and 75.0%.^3^ Furthermore, coverage has not been homogeneous in Brazil and studies report profound inequalities in vaccination coverage among infants, according to the socioeconomic stratum and region of residence of their families.^4-6^ The drop in coverage seen in Brazil since 2016 may be explained by the increase in vaccine hesitancy aggravated by anti-vaccine movements and campaigns disseminating false information, underfunding of the SUS, local governments not prioritizing vaccination and the worsening of the population’s socioeconomic status.^7-9^

Vaccination coverage is a fundamental indicator of the performance of immunization programs. However, the importance of timely adherence to the vaccination schedule must be emphasizd, as delays can lead to immunization failures. With regard to individual protection, timely vaccination guarantees maximum effectiveness of immunobiological products and indirectly, through herd immunity, protects the community from vaccine-preventable diseases. Late vaccination can increase people’s vulnerability to vaccine-preventable diseases. Furthermore, studies indicate that delay is associated with greater risk of abandoning the vaccination schedule.^10-12^ Delays in vaccination are one of the components of vaccine hesitancy, which is characterized by a continuum between those who refuse certain vaccines and those who refuse all of them, including those who delay vaccination.^9^

The potential impact of delayed vaccinations on protection against vaccine-preventable diseases in infants, who represent an important vulnerable group within the population, reinforces the importance of updating knowledge on the subject.

The objective of this study was to evaluate vaccination coverage and delay in vaccine dose administration in infants in six municipalities in the Southern region of Brazil.

## METHODS

### Study design 

This is a population-based survey. Vaccination data were obtained longitudinally, by analyzing vaccination card records for each child included in the sample.^1^

### Background 

The south Brazilian cities included in the National Vaccination Coverage Survey – 2020 (INCV 2020) were the region’s three state capitals with populations (inhabitants) estimated for 2020 (Curitiba ‒ 1,751,907, Florianópolis ‒ 508,826 and Porto Alegre ‒ 1,488,252) and three municipalities in the interior regions of the same states with more than 100,000 inhabitants (Londrina – 506,701, Joinville – 604,708 and Rio Grande – 211,965), respectively. In 2021, Londrina and Rio Grande had a Municipal Development Index (MDI) classified as “high”, while the other cities had a “very high” MDI.^13^

### Participants

The target population was comprised of 129,505 infants born live to mothers residing in the municipalities of the Southern region of Brazil participating in the survey, according to the Live Birth Information System (SINASC), which included infants born in 2017 and 2018 residing in the three state capitals - Curitiba (PR) 44,857 live births, Florianópolis (SC) 12,723 live births and Porto Alegre (RS) 36,069 live births ‒ and in three cities in the interior region of the states ‒ Londrina (PR) 14,118 live births, Joinville (SC) 16,260 live births and Rio Grande (RS) 5,478 live births.^1,13^

The sampling procedure considered the representativeness of infants, according to socioeconomic strata, and followed the 2010 Census tracts, with a design effect 1,4, hypothetical population (1 million live births), estimated coverage prevalence (70%), estimation error (5%) and confidence interval (95%) resulting in a sample of 452 infants per survey. In each city, a sample of one to four surveys was proposed according to the number of live births registered on the SINASC for the years 2017 and 2018.^1^

### Variables

The outcomes analyzed were coverage in the Southern region for each vaccine included on the PNI vaccination schedule and for the set of vaccines recommended up to 12 months old (full basic schedule): BCG, hepatitis B (HB), three doses of 5-in-1 vaccine (diphtheria, tetanus, pertussis (DTP)+*haemophilus influenzae* type B+hepatitis B) and three doses of inactivated poliovirus vaccine (IPV), two doses against rotavirus (ROTA), two doses of meningococcal c (MenC) and pneumococcal conjugate 10-valent (PCV10) vaccines; and up to 24 months old (full schedule at 24 months): basic schedule vaccines plus two doses of measles, mumps and rubella (MMR) vaccine, one dose each of hepatitis A (HA) vaccine, varicella vaccine and attenuated oral poliovirus vaccine (OPV); and one booster dose each of diphtheria, tetanus and pertussis (DTP), MenC and PCV10 vaccines. Vaccines administered exclusively in private vaccination services compatible with the schedules indicated by the PNI were included in the assessment of the vaccination schedule: doses of diphtheria, tetanus and acellular pertussis (DTPa), acellular 5-in-1 (DTPa+*haemophilus influenzae* type B+IPV), acellular hexavalent (acellular 5-in-1 + HB), hepatitis A and B vaccine, meningococcal ACWY, meningococcal B, second dose against measles, mumps, rubella and varicella (MMRV) and a third dose each of rotavirus vaccine and pneumococcal conjugate 13-valent vaccine. Yellow fever vaccine was excluded from the study, as it was not part of the children’s schedule in some participating municipalities. We defined the full basic coverage (up to 12 months), full coverage between 12 and 24 months and full coverage at 24 months indicators (full basic coverage, full 12 and 24 coverage, full coverage at 24). Coverage was evaluated according to doses administered and doses administered on time, as per the following formulae:


$$\text{Coverage of vaccine X}=\frac{\text{No. of infants receiving dose of vaccine X}}{\text{No. of infants in the sample}}\times 100$$

$$\text{Full basic coverage (doses administered)}=\frac{\text{No. of infants receiving all doses scheduled by 12 months}}{\text{No. of infants in the sample}}\;\times \;100$$

$$\text{Full 12 and 24 coverage (doses administered)}=\frac{\text{No. of infants receiving all doses scheduled between 12 and 24 months}}{\text{No. of infants in the sample}}\times \;100$$

$$\text{Full coverage at 24 (doses administered)}=\frac{\text{No. of infants receiving all doses scheduled by 24 months}}{\text{No. of infants in the sample}}\times \;100$$

$$\text{​Full basic coverage (doses administered on time)}=\frac{\text{No. of infants receiving all doses scheduled in the first year of life administered on time}}{\text{No. of infants in the sample}}\times \;100$$

$$\text{Full 12 and 24 coverage (doses administered on time)}=\frac{\text{No. of infants receiving all doses scheduled between 12 and 24 months administered on time}}{\text{No. of infants in the sample}}$$

$$\text{​Full coverage at 24 (doses administered on time)}=\frac{\text{No. of infants receiving all doses scheduled by 24 months administered on time}}{\text{No. of infants in the sample}}\times \;100$$

Administered dose was defined as record of vaccination by 24 months, regardless of the time it was administered. With regard to the definition of doses administered on time, we used the INCV 2020 criterion which took on time administration to be the interval between the PNI recommendation for vaccine administration plus 30 days. Delay was defined as: BCG and HB ≥ 31 days of life; 2^nd^ dose of PCV10 and ROTA ≥ 152 days; 2^nd^ dose of MenC ≥ 182 days; 3^rd^ dose of 5-in-1 and IPV ≥ 213 days; PCV10 booster, MenC and 1^st^ dose of ≥ 395 days; dose of HA, 2^nd^ dose of MMR, 1^st^ OPV booster, 1^st^ DTP booster and 1^st^ dose of varicella ≥ 487 days.^1^ Vaccines administered in private services compatible with the PNI vaccination schedule were also included. Delay time was categorized into: delay of less than three months (up to 90 days); from three months to five months and 29 days (from 91 to 180 days) and six months delay or more (181 days or more). For this indicator, we assessed the delay in the Southern region for all doses of vaccines defined on the schedule for administration by 24 months.

Full coverage of doses administered and doses administered on time in the Southern region was described according to: socioeconomic stratum of the census tract of residence (A, B, C and D), maternal schooling (up to eight years of study, nine to 12 years of study, 13 to 15 years of study, 16 years or more), maternal age (< 20 years, 20 to 34 years, 35 years and more), maternal race/skin color (White, Black, mixed race, Asian, Indigenous), mother having a partner (yes, no), number of children (1, 2, 3+), use of a private service at some point for vaccination (yes, no) and children attending nursery/daycare (yes, no). The data used to classify the census tracts of residence were: average income of heads of household, proportion of literate heads of household and proportion of heads of household with income greater than or equal to 20 minimum wages, whereby A was the stratum with the best socioeconomic condition and D was the stratum with the poorest.^1^

### Data sources 

For this study we used the INCV 2020 Southern region anonymized database, which we accessed from September to December 2023. INCV 2020 data was collected through electronic devices used in households, based on addresses held on the SINASC and also by means of active tracking in selected clusters, between September 2020 and March 2022. In order to identify the doses administered, each vaccination card was photographed and the data were subsequently input to the database. Socioeconomic, behavioral and vaccine hesitancy information was collected by means of administering a structured questionnaire. More details about INCV 2020 are given in previous publications.^1,13^

### Statistical methods 

Vaccine coverage, percentage delays in vaccination and confidence intervals were calculated using Stata® version 17, by means of its survey analysis module. Given that the survey sample was stratified and clustered by census tract based on the socioeconomic stratum of area of ​​residence, weighted analysis was carried out with sample weighting for each of the households interviewed, which also enabled avoiding possible biases resulting from sample losses.^1^

We used 95% confidence intervals in order to identify differences between coverage levels.

### Ethical considerations 

The survey was approved by the Human Research Ethics Committee of the *Instituto de Saúde Coletiva* of the *Universidade Federal da Bahia*, as per Opinion No. 3.366.818, on June 4^th^ 2019, and Certificate of Submission for Ethical Appraisal No. 4306919.5.0000.5030; and by the Human Research Ethics Committee of the *Irmandade da Santa Casa de São Paulo*, as per Opinion No. 4.380.019, on November 4^th^ 2020, and Certificate of Submission for Ethical Appraisal No. 39412020.0.0000.5479. The guardians of the infants gave their informed consent when the primary data were collected. 

## RESULTS

The final Southern region sample was composed of 4,681 infants born in 2017 and 2018, with 1,383 born in Porto Alegre (RS), 1,192 in Curitiba (PR), 739 in Florianópolis (SC), 460 in Joinville (SC), 455 in Londrina (PR) and 452 in Rio Grande (RS). Sample loss was 14% and occurred only in the municipalities of Curitiba (34%) and Florianópolis (18%), and was more frequent in stratum A.

The sample distribution according to socioeconomic profile is shown in Table 1. Participants were mostly from strata C and D. Regarding the profile of the mothers, we found higher proportions of maternal schooling with more than 16 years of study, age above 35 years, White race/skin color, having a partner and only one child. In the sample studied, 36.9% of infants used a private vaccination service at some time.

**Table 1:** Profile of the sample and full vaccination coverage at 24 months of administered doses and on-time doses, according to socioeconomic characteristics, among infants born in municipalities of the Southern region of Brazil, 2020-2022 (n = 4,681)

**Variables**	Categories	**Sample** ^a^		**Vaccination coverage (%)**			
		**n**	**%**	**Doses administered**		**Doses on time**	
				**(95%CI)**		**(95%CI)**	
**Socioeconomic stratum of area of residence**	A	893	19.1	60.6	(53.6;67.1)	2.9	(1.6;5.5)
	B	1,042	22.3	66.5	(55.6;75.9)	4.8	(1.8;12.1)
	C	1,373	29.3	66.7	(59.1;73.5)	2.1	(1.3;3.4)
	D	1,373	29.3	70.9	(65.1;76.2)	5.0	(2.9;8.3)
**Maternal schooling (years)**	≤ 12	898	19.5	61.9	(54.3;69.1)	2.5	(0.1;6.6)
	13-15	1,534	33.3	71.5	(66.0;76.4)	4.5	(2.6;7.8)
	16 or more	2,170	47.2	68.5	(61.6;74.6)	4.1	(2.1;7.9)
**Maternal** **age**	< 20 years	59	1.3	32.9	(8.7;71.8)	0.0	-
	20-34 years	2,251	48.2	68.5	(63.9;72.8)	3.1	(2.0;4.9)
	≥ 35 years	2,358	50.5	69.9	(64.7;74.6)	5.1	(2.8;9.2)
**Maternal race/skin color**	White	3,603	78.4	70.3	(66.1;74.2)	4.1	(2.7;6.3)
	Black	403	8.8	64.3	(54.3;73.2)	5.7	(1.5;9.4)
	Brown-skinned	552	12.0	60.1	(46.9;71.9)	1.8	(0.9;3.6)
	Asian	35	0.8	70.8	(44.4;88.1)	0.3	(0.1;2.0)
	Indigenous	2	0.0	38.0	(3.7;90.8)	0.0	-
**Mother has a partner**	Yes	3,740	81.5	70.3	(66.1;74.3)	3.2	(2.3;4.5)
	No	847	18.5	61.0	(53.6;67.9)	6.6	(2.6;16.0)
**Number of** **children**	1	1,925	41.1	69.9	(63.7;75.5)	5.7	(3.4;9.5)
	2	1,846	39.4	65.8	(59.9;71.3)	3.5	(1.8;7.0)
	3+	911	19.5	68.7	(62.3;74.5)	1.2	(0.6;2.5)
**Use of private service** ^b^	Yes	1,719	36.9	67.8	(59.7;75.0)	4.5	(2.2;9.2)
	No	2,935	63.1	68.1	(63.6;72.3)	3.6	(2.4;5.5)
**Attends nursery/daycare**	Yes	2,764	59.1	68.8	(63.7;73.5)	4.7	(2.8;7.9)
	No	1,912	40.9	67.1	(62.0;71.9)	2.8	(1.7;4.7)

a) Categories with unknown values or values that were not informed are not shown; b) For any vaccine.

**Figure 1:**
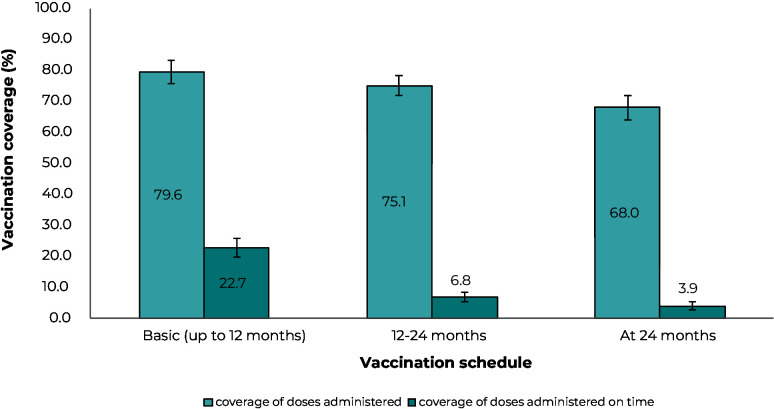
Vaccination coverage of doses administered and doses administered on time, according to the basic vaccination schedule (at 12 months), from 12 to 24 months and at 24 months, among infants born in municipalities in the Southern region of Brazil, 2020-2022

Coverage of doses administered and doses administered on time for the set of municipalities participating in INCV 2020 in the Southern region of Brazil is shown in Figure 1. Full vaccination coverage at 24 months for doses administered was 68.0% (95%CI 63.9;71.8%), while for doses administered on time it was 3.9% (95%CI 2.7%;5.7%). The values ​​for full basic coverage and full coverage at 12 and 24 months are similar in terms of doses administered, being 79.6% (95%CI 76.1;83.2%) and 75.1% (95%CI 72,0;78.4%), respectively. With regard to coverage on time, there was a higher proportion of doses administered up to 12 months (22.7%; 95%CI 19.5;26.0%), when compared to vaccines indicated for 12 and 24 months (6.8%; 95%CI 5.3;8.6%). No differences were found in full coverage for doses administered and doses administered on time according to the variables analyzed (Table 1), except in the comparison between one child and three children or more for doses administered on time.

**Figure 2:**
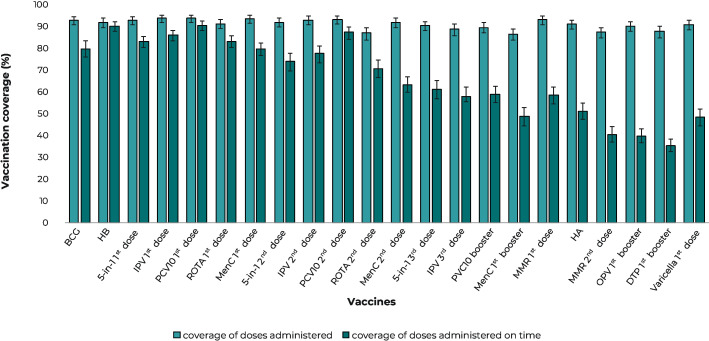
Vaccination coverage of doses administered and doses administered on time, for vaccines indicated up to 24 months, according to doses, among infants born in municipalities in the Southern region of Brazil, 2020-2022

When analyzing coverage of doses administered according to each vaccine (Figure 2), we found that most vaccines achieved or exceeded 90% coverage. The following vaccines had coverage below 90%: ROTA 2^nd^ dose (87%), IPV 3^rd^ dose (89%), MenC 1^st^ booster (87%), MMR 2^nd^ dose (87%) and DTP 1^st^ booster (88%). When analyzing doses administered on time, we found that only two vaccines achieved coverage equal to or greater than 90% (HB and PCV10 1^st^ dose) and that coverage decreased as the age at which the vaccine is indicated increased, being below 50% for the MenC 1^st^ booster, MMR 2^nd^ dose, OPV 1^st^ booster, DTP 1^st^ booster and varicella 1^st^ dose. 

**Figure 3:**
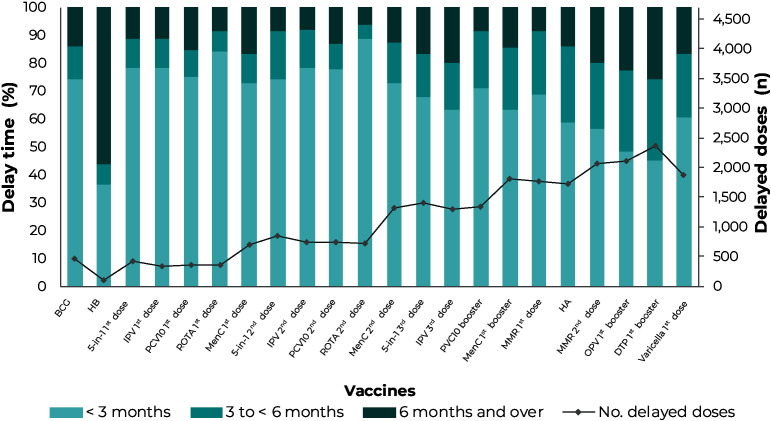
Delay time of vaccines administered among infants born in municipalities in the Southern region of Brazil, 2020-2022

Regarding vaccination delay time (Figure 3), there was an increase in the number of delayed doses as the age at which the vaccine is indicated increased, as well as in subsequent doses of schedules with more than one dose and/or booster. The same situation occurred in the proportion of doses administered more than three months late, reaching higher proportions for the first DTP and OPV boosters. The HB vaccine is an exception, as 56% of doses were administered six months late or more, highlighting, however, that only 110 infants received this vaccine late.

## DISCUSSION

The main contribution of this study is to provide recent information on the occurrence of vaccination delays among infants in Brazil. Analysis of coverage of doses administered and doses administered on time in the Southern region for infants born before the COVID-19 pandemic, revealed administered dose coverage of less than 90% for five vaccines, with worrying results for on-time doses which achieved less than 50% for five of the vaccines.

Full coverage of doses administered, considering all vaccines indicated up to 24 months, approximately 70%, although higher than that found for Brazil as a whole in the same survey (59.9%; 95%CI 58.3;61,5),^1^ demonstrates the challenge of achieving vaccination coverage targets. A more worrying situation lies in the fact that only one in five infants received their vaccines on time by 12 months and less than 10% completed the routine vaccination schedule on time by 24 months. National and international studies demonstrate a drop in coverage^1,8,15-19^ and highlight concern regarding greater vulnerability to the occurrence of vaccine-preventable diseases.^14^ When evaluating each vaccine comprising the vaccination schedule, there is clearly a progressive drop in the coverage of administered doses with lower coverage for subsequent vaccine doses (D2 and D3) and booster doses. Studies in other Brazilian states that evaluated vaccines that have second doses and/or booster doses, showed greater drops in the coverage of subsequent doses and/or booster doses.^17,18^

Coverage was lower for vaccination on time, especially for subsequent doses, boosters and doses administered after 12 months old. Research carried out in a municipality in the interior region of São Paulo state with children born in 2012 showed a large proportion of vaccination delays for vaccines indicated in the second year of life, with 28.9% of children vaccinated on time with the second dose of MMR and DTP, OPV and pneumococcal boosters.^19^ A study based on the 2013 Brazilian National Health Survey that assessed prevalence of delay in administering the three doses of tetravalent vaccine (DTP+ *haemophilus influenzae* type B) among infants aged 12-23 months, found that there was 14.8% delay for the first dose, 28.8% for the second dose and 45.4% for the third dose.^11^ Other countries also show an increase in vaccination delays as the doses progress.^20-24^ These findings indicate the need for booster guidance regarding completion of the vaccination schedule, including boosters at the appropriate time, especially in the second year of life when the frequency of delay and abandonment is greater.

Although on-time coverage according to socioeconomic profile shows higher proportions among infants of mothers with higher education levels, with fewer children, who had a partner and lived in stratum D areas, and whose children attended daycare/nursery, no statistically significant differences were identified regarding coverage of doses administered and doses administered on time, contrary to previous studies that demonstrate association with socioeconomic factors, such as schooling and income. Whereas studies carried out in the North and Northeast regions of Brazil indicated poorer coverage in strata with lower income and schooling,^15,16^ in São Paulo coverage was found to increase as socioeconomic level decreased.^25^ Assessment of tetravalent vaccination delay in 2013, in Brazil, showed a higher proportion of delay in children of mixed race/skin color, belonging to poorer families, resident in rural areas and the Northern region of the country.^11^ Data from 2008 to 2018 on children vaccinated in England showed an inverse relationship between timely vaccination and socioeconomic level, in addition to differences according to the regions of the country.^20^

When evaluating length of delay, differences were found according to vaccine doses, with delays being lower for the first and second doses and higher for third doses, boosters and doses administered from 12 months onwards. In the case of the DTP and OPV boosters, the delay was greater than 60% and one in every four infants with delayed vaccinations were vaccinated more than six months after the recommended date. Delay in vaccination leads to prolonged exposure of infants to vaccine-preventable diseases, more common in childhood, and also increases the risk of not completing the routine vaccination schedule.^13,19^ For example, standing out due to delays in vaccination is the occurrence of measles outbreaks worldwide, despite high global coverage.^19^ A study in a hospital in Saudi Arabia, conducted in 2018, analyzing the vaccines in that country’s childhood schedule, revealed that vaccination of 59.1% of children was delayed by at least one month.^26^ In Montana, in the United States, only 38.0% of infants evaluated from 2015 to 2019 received all doses of the vaccine on time.^27^ When evaluating data from the 2012 national survey in the United States, Kurosky et al. ^28^ found that in addition to low levels of timely vaccination, there were prolonged delays of up to seven months. 

The high proportions of vaccination delays point to the need for vaccination services, especially in primary care, to reinforce actions to ensure vaccination on time. The use of upcoming vaccine date reminder strategies is recommended in the United States by the Task Force on Community Preventive Services to increase immunization rates.^29^ With the progress achieved by communication technologies, it is possible to send messages via e-mail and cell phone, in addition to bolstering guidance given by childcare professionals and vaccination room staff in order to increase confidence in vaccines and the importance of maintaining keeping vaccinations up to date.^27,29,30^ Furthermore, expanding the coverage of the Family Health Strategy with complete teams and the role of Community Health Agents in active tracking are also important measures. Reducing barriers to access, whether material barriers related to families, urban barriers or those linked to health services, should be a priority for municipal health service managers in Brazil.

Some limitations of this study need to be highlighted. There were sample losses, especially in the municipalities of Curitiba and Florianópolis, also influenced by the time at which the data were collected, which coincided with the period of social distancing measures due to the COVID-19 pandemic. However, territorial expansion strategies for data collection and the use of sampling weights, considering those groups in which there were greater losses, minimized the effects of this sample loss. It is noteworthy that the data analyzed are related to the Southern region and cannot be generalized to the rest of Brazil. In the Southern region sample, there was a higher proportion of mothers with 16 years of schooling or more, aged over 34 years and of White race/skin color, these being characteristics distinct from those found for the profile of INCV 2020 interviewees nationwide.^1^

Considering that this study described the panorama found in six cities in the south of Brazil, it is important that new studies be conducted with the aim of understanding the reality of other regions of the country as well as the national context, not only regarding vaccination coverage, but also assessing vaccination delay and associated factors. 

This study also points out that in addition to ensuring vaccination, it is extremely important to follow the vaccination schedule correctly. Given that coverage is generally assessed at the end of the first and second years of life, several months after the recommended age for vaccination, it is essential that vaccination monitoring systems check whether vaccines are being administered at the recommended ages, with the adoption of strategies that reinforce routine vaccination to prevent vaccine delays, this being an essential primary care action. Guidance to children’s guardians on the need to keep vaccinations up to date and the adoption of reminder strategies for upcoming doses must be reinforced, rather than solely concentrating efforts on campaigns to track down defaulters.
