# Supplementary material for: Vaccination coverage and delay in vaccination of infants born in 2017 and 2018 in municipalities in the Southern region of Brazil: National Vaccination Coverage Survey 2020
Source: Epidemiol Serv Saude. 2024 Oct 21;33(spe2):e20231206. doi: 10.1590/S2237-96222024v33e20231206.especial2.en (PMC11493396; doi:10.1590/S2237-96222024v33e20231206.especial2.en)
Supplement: Translation

# Translation: Cobertura vacinal e atraso na vacinação de lactentes nascidos em 2017 e 2018 em municípios da região Sul do Brasil: Inquérito Nacional de Cobertura Vacinal

**DOI:** 10.1590/S2237-96222024v33e20231206.especial2.pt

**Authors:** Karin Regina Luhm, Antonio Fernando Boing, Sotero Serrate Mengue, Neiva de Souza Daniel, Tainá Ribas Mélo, Raquel Jaqueline Farion, Ana Paula França, José Cássio de Moraes, Grupo ICV 2020, Adriana Ilha da Silva, Alberto Novaes Ramos, Ana Paula França, Andrea de Nazaré Marvão Oliveira, Antonio Fernando Boing, Carla Magda Allan Santos Domingues, Consuelo Silva de Oliveira, Ethel Leonor Noia Maciel, Ione Aquemi Guibu, Isabelle Ribeiro Barbosa Mirabal, Jaqueline Caracas Barbosa, Jaqueline Costa Lima, José Cássio de Moraes, Karin Regina Luhm, Karlla Antonieta Amorim Caetano, Luisa Helena de Oliveira Lima, Maria Bernadete de Cerqueira Antunes, Maria da Gloria Teixeira, Maria Denise de Castro Teixeira, Maria Fernanda de Sousa Oliveira Borges, Rejane Christine de Sousa Queiroz, Ricardo Queiroz Gurgel, Rita Barradas Barata, Roberta Nogueira Calandrini de Azevedo, Sandra Maria do Valle Leone de Oliveira, Sheila Araújo Teles, Silvana Granado Nogueira da Gama, Sotero Serrate Mengue, Taynãna César Simões, Valdir Nascimento, Wildo Navegantes de Araújo

**Affiliations:** 1 Universidade Federal do Paraná, Departamento de Saúde Coletiva, Curitiba, PR, Brasil; 2 Universidade Federal de Santa Catarina, Departamento de Saúde Pública, Florianópolis, SC, Brasil; 3 Universidade Federal do Rio Grande do Sul, Programa de Pós-Graduação em Epidemiologia, Porto Alegre, RS, Brasil; 4 Universidade Federal do Paraná, Programa de Pós-Graduação em Saúde Coletiva, Curitiba, PR, Brasil; 5 Universidade Federal do Paraná, Pós-Graduação e Graduação em Saúde Coletiva, Curitiba, PR, Brasil; 6 Pontifícia Universidade Católica do Paraná, Escola de Medicina e Ciências da Vida, Curitiba, PR, Brasil; 7 Faculdade de Ciências Médicas da Santa Casa de São Paulo, Departamento de Saúde Coletiva, São Paulo, SP, Brasil; Universidade Federal do Espírito Santo, Vitória, ES, Brasil; Universidade Federal do Ceará, Departamento de Saúde Comunitária, Fortaleza, CE, Brasil; Faculdade Ciências Médicas Santa Casa de São Paulo, São Paulo, SP, Brasil; Secretaria de Estado da Saúde do Amapá, Macapá, AP, Brasil; Universidade Federal de Santa Catarina, SC, Brasil; Organização Pan-Americana da Saúde, Brasília, DF, Brasil; Instituto Evandro Chagas, Belém, PA, Brasil; Faculdade de Ciências Médicas Santa Casa de São Paulo, Departamento de Saúde Coletiva, São Paulo, SP, Brasil; Universidade Federal do Rio Grande do Norte, Natal, RN, Brasil; Universidade Federal do Ceará, Departamento de Saúde Comunitária, Fortaleza, CE, Brasil; Universidade Federal de Mato Grosso, Cuiabá, MT, Brasil; Universidade Federal do Paraná, Curitiba, PR, Brasil; Universidade Federal de Goiás, Goiânia, GO, Brasil; Universidade Federal do Piauí, Teresina, PI, Brasil; Universidade de Pernambuco, Faculdade de Ciências Médicas, Pernambuco, PE, Brasil; Instituto de Saúde Coletiva, Universidade Federal da Bahia, Salvador, BA, Brasil; Secretaria de Estado da Saúde de Alagoas, Maceió, AL, Brasil; Universidade Federal do Acre, Rio Branco, AC, Brasil; Universidade Federal do Maranhão, Departamento de Saúde Pública, São Luís, MA, Brasil; Universidade Federal de Sergipe, Aracaju, SE, Brasil; Secretaria Municipal de Saúde, Boa Vista, RR, Brasil; Fundação Oswaldo Cruz, Mato Grosso do Sul, Campo Grande, MS, Brasil; Fundação Oswaldo Cruz, Escola Nacional de Saúde Pública Sergio Arouca, Rio de Janeiro, RJ, Brasil; Universidade Federal do Rio Grande do Sul, Porto Alegre, RS, Brasil; Fundação Oswaldo Cruz, Instituto de Pesquisa René Rachou, Belo Horizonte, MG, Brasil; Secretaria de Desenvolvimento Ambiental de Rondônia, Porto Velho, RO, Brasil; Universidade de Brasília, Brasília, DF, Brasil

**Keywords:** Cobertura Vacinal, Vacinas, Inquéritos Epidemiológicos, Hesitação Vacinal, Atraso Vacinal

## Abstract

**Objetivo:**

Avaliar as coberturas vacinais e o atraso nas doses de vacinas em lactentes em seis municípios da região Sul do Brasil.

**Metodologia:**

Inquérito Nacional de Cobertura Vacinal 2020, com lactentes nascidos vivos em 2017 e 2018, realizado entre setembro de 2020 e março de 2022. Foram avaliadas as coberturas de doses aplicadas, doses em dia e o tempo de atraso da aplicação.

**Resultados:**

Para 4.681 lactentes analisados, as coberturas para vacinas indicadas até os 24 meses foram de 68,0% (IC_95_% 63,9;71,8) para doses aplicadas e 3,9% (IC_95_% 2,7;5,7) para doses em dia. A maioria das aplicações em atraso foi ≤ 3 meses. Para alguns reforços, 25% das aplicações atrasaram ≥ 6 meses.

**Conclusão:**

Além da busca de faltosos às vacinas, são necessárias estratégias para estímulo ao cumprimento do esquema de vacinação nas idades preconizadas.

## Contribuições do estudo

**Principais resultados:**

A cobertura vacinal para o conjunto de vacinas indicadas até os 24 meses foi 68,0%, e 3,9% para doses em dia. O tempo de atraso para algumas doses ultrapassou seis meses em até 25% dos lactentes com atraso.

**Implicações para os serviços:**

O monitoramento da aplicação das vacinas nas idades preconizadas faz-se necessário, com a adoção de estratégias que reforcem a vacinação de rotina para prevenção do atraso e abandono vacinal.

**Perspectivas:**

Atenção primária na vigilância e atenção aos lactentes precisa reforçar as ações para garantir a vacinação em dia. Estudos para aprofundar o conhecimento do atraso vacinal, determinantes e estratégias para a redução são necessários.

## INTRODUÇÃO

Importante ação de saúde pública para a prevenção de doenças, a vacinação reduziu significativamente a morbimortalidade das enfermidades imunopreveníveis no Brasil. Impulsionada pelo Programa Nacional de Imunização (PNI), a vacinação é uma ação estratégica do Sistema Único de Saúde (SUS) no país. Os imunizantes são oferecidos de maneira universal e gratuita, segundo um calendário vacinal de rotina para todo o ciclo de vida da população.^1^

O êxito das ações do PNI deve-se ao seu grande alcance populacional (todos os territórios e classes sociais), à sua alta taxa de cobertura vacinal ‒ garantindo a limitação da disseminação dos agentes causadores de doenças imunopreveníveis ‒, à qualidade dos insumos aplicados e dos procedimentos de aplicação, à acessibilidade aos serviços e ao combate às barreiras socioeconômicas e culturais existentes.^1^

Apesar da relevância e do impacto positivo do PNI ao longo de cinco décadas, o Brasil apresentou, entre 2016 e 2021, acentuada queda na cobertura de diferentes imunobiológicos. Em 2021, estavam disponíveis 14 vacinas no calendário vacinal de rotina para menores de 2 anos de idade,^2^ porém o país não atingiu o quantitativo de 75% de cobertura em nenhum imunobiológico naquele ano. A cobertura da vacina pneumocócica oscilou, entre 2016 e 2021, de 95,0% para 74,8%, da meningocócica, de 91,7% para 72,2%, e a bacilo de Calmette-Guérin (BCG), de 95,6% para 75,0%.^3^ Além disso, as coberturas não têm sido homogêneas no país e estudos relatam profundas desigualdades na cobertura vacinal em lactentes, de acordo com o estrato socioeconômico e com a região de residência das famílias.^4-6^ A queda na cobertura observada no Brasil desde 2016 pode ser explicada pelo aumento da hesitação vacinal amplificada pelos movimentos antivacinas e campanhas de disseminação de informações falsas, pelo subfinanciamento do SUS, não priorização de governos em relação à vacinação e ao agravamento da situação socioeconômica da população.^7-9^

A cobertura vacinal é um indicador fundamental do desempenho dos programas de imunização. Contudo, deve-se destacar a importância da adesão vacinal oportuna ao calendário de vacinação, visto que atrasos podem gerar falhas na imunização. Na proteção individual, a vacinação oportuna garante a máxima efetividade dos imunobiológicos e indiretamente, através da imunidade coletiva, protege a comunidade de doenças imunopreveníveis. Vacinas aplicadas com atraso podem aumentar a vulnerabilidade dos indivíduos para doenças imunopreveníveis. Além disso, estudos apontam que o atraso está associado ao maior risco de abandono do esquema vacinal.^10-12^ Atrasos na vacinação representam um dos componentes da hesitação vacinal, que é caracterizada por um *continuum* entre aqueles que recusam algumas vacinas e os que recusam todas, incluindo os que atrasam a vacinação.^9^

O potencial impacto do atraso vacinal na proteção contra doenças imunopreveníveis em lactentes, que representam um importante grupo vulnerável da população, reforça a importância da atualização do conhecimento sobre a temática.

O objetivo deste estudo foi avaliar a cobertura vacinal e o atraso nas doses de vacinas em lactentes em seis municípios da região Sul do Brasil.

## MÉTODOS

### Desenho de estudo

Trata-se de inquérito de base populacional. Os dados de vacinação foram obtidos de forma longitudinal, a partir da avaliação dos registros da caderneta de vacinação para cada criança incluída na amostra.^1^

### Contexto

As cidades da região Sul incluídas no Inquérito Nacional de Cobertura Vacinal – 2020 (INCV 2020) foram as três capitais com populações estimadas (habitantes) para 2020 (Curitiba ‒ 1.751.907, Florianópolis ‒ 508.826 e Porto Alegre ‒ 1.488.252) e três municípios do interior com mais de 100 mil habitantes (Londrina – 506.701, Joinville – 604.708 e Rio Grande – 211.965). Londrina e Rio Grande possuíam Índice de Desenvolvimento Municipal, em 2010, classificado como “alto”, e os demais, “muito alto”.^13^

### Participantes

A população-alvo foi de 129.505 nascidos vivos de mães residentes nos municípios da região Sul do Brasil participantes do inquérito, de acordo com o Sistema de Informação sobre Nascidos Vivos (Sinasc) que incluiu lactentes nascidos em 2017 e 2018 residentes nas três capitais ‒ Curitiba (PR), 44.857 nascidos vivos, Florianópolis (SC), 12.723 nascidos vivos e Porto Alegre (RS), 36.069 nascidos vivos ‒ e em três cidades do interior dos estados ‒ Londrina (PR), 14.118 nascidos vivos, Joinville (SC), 16.260 nascidos vivos e Rio Grande (RS), 5.478 nascidos vivos.^1,13^

O procedimento amostral contemplou a representatividade dos lactentes, segundo estratos socioeconômicos, e seguiu os recortes dos setores censitários do Censo de 2010, com um efeito do desenho 1,4, população hipotética (1 milhão de nascidos vivos), prevalência estimada de cobertura (70%), erro de estimativa (5%) e intervalo de confiança (95%), resultando numa amostra de 452 lactentes por inquérito. Em cada cidade, foi proposta uma amostra de um a quatro inquéritos, de acordo com o número de nascidos vivos registrados Sinasc em 2017 e 2018.^1^

### Variáveis

Os desfechos analisados foram a cobertura na região Sul para cada vacina prevista no calendário vacinal do PNI e para o conjunto de vacinas indicadas até os 12 meses (esquema básico completo): BCG, hepatite B (HB), três doses de pentavalente (tríplice bacteriana+*haemophilus influenzae* tipo B+hepatite B – penta) e de vacina inativada de poliovírus (VIP), duas doses contra rotavírus (ROTA), duas doses da MenC- e pneumocócica conjugada 10 valente (VPC10) e até os 24 meses (esquema completo aos 24 meses): vacinas do esquema básico e duas doses de tríplice viral (sarampo, rubéola e caxumba ‒ SCR), uma dose de hepatite A (HA), varicela e vacina oral de poliovírus atenuados (VOP); e uma dose de reforço com tríplice bacteriana (difteria, tétano e pertússis, DTP), MenC e VPC10. Foram incluídas na avaliação do esquema vacinal as vacinas aplicadas exclusivamente nos serviços privados de vacinação e que são compatíveis com os esquemas indicados pelo PNI: qualquer dose de tríplice bacteriana acelular (DTPa), pentavalente acelular (DTPa+*haemophilus influenzae* tipo B+VIP) , hexavalente acelular (pentavalente acelular + HB) , vacina hepatite A e B , meningocócica ACWY, meningocócica B, segunda dose de tetraviral (SCRV ‒ contra sarampo, rubéola caxumba e varicela) e terceira dose de vacina rotavírus e pneumocócica conjugada 13 valente. A vacina para febre amarela foi excluída do estudo, pois não fazia parte do calendário infantil em alguns municípios participantes. Foram definidos os indicadores cobertura básica completa até 12 meses, cobertura completa entre 12 e 24 meses e cobertura completa aos 24 meses (cobertura básica completa, cobertura completa entre 12 e 24 meses, cobertura completa aos 24). As coberturas foram avaliadas segundo doses aplicadas e doses aplicadas em dia segundo as fórmulas:


$$\text{Cobertura segundo vacina X}=\frac{(\text{N° lactentes com a dose da vacina X}}{\text{N° lactentes da amostra}}\times 100$$

$$\text{Cobertura básica completa (doses aplicadas)}=\frac{\text{N° lactentes com todas as doses previstas até os 12 meses}}{\text{N° lactentes da amostra}}\times 100$$

$$\text{Cobertura completa entre 12 e 24 meses (doses aplicadas)}=\frac{\text{N° lactentes com todas as doses previstas de 12 aos 24 meses}}{\text{N° lactentes da amostra}}\times 100$$

$$\text{Cobertura completa aos 24 meses (doses aplicadas)}=\frac{\text{N° de lactentes com todas as doses previstas até os 24 meses}}{\text{N° de lactentes da amostra}}\times 100$$

$$\text{Cobertura básica completa (doses em dia)}=\frac{\text{N° de lactentes com todas as doses previstas no primeiro ano de vida aplicadas em dia}}{\text{N° de lactentes da amostra}}\times 100$$

$$\text{Cobertura completa entre 12 e 24 meses (doses em dia)}=\frac{\text{N° de lactentes com todas as doses previstas dos 12 aos 24 meses aplicadas em dia}}{\text{N° de lactentes da amostra}}$$

$$\text{Cobertura completa aos 24 meses (doses em dia)}=\frac{\text{N° de lactentes com todas as doses previstas até os 24 meses aplicadas em dia}}{\text{N° de lactentes da amostra}}\times 100$$

A definição de dose aplicada foi o registro de vacina até 24 meses, independentemente do período de aplicação. Para dose aplicada em dia, foi utilizado o critério do INCV 2020, que considerou em dia o intervalo entre a recomendação do PNI para a aplicação da vacina acrescida de 30 dias. O atraso foi definido: para BCG e HB ≥ 31 dias de vida; 2ª dose de VPC10 e ROTA ≥ 152 dias; 2ª dose de MenC ≥ 182 dias; 3ª dose da penta e VIP ≥ 213 dias; reforço VPC10, MenC e 1ª dose de ≥ 395 dias; dose de HA, 2ª dose de SCR, 1º reforço VOP, 1º reforço DTP e 1º dose de varicela ≥ 487 dias.^1^ Foram também incluídas as vacinas privadas compatíveis com o esquema do PNI. O tempo de atraso foi categorizado em: atraso inferior a três meses (até 90 dias); de três meses a cinco meses e 29 dias (de 91 a 180 dias) e de seis meses ou mais de atraso (181 dias ou mais). Para este indicador, foi avaliado o atraso da região Sul para o conjunto de todas as doses definidas no calendário até os 24 meses.

A cobertura completa de doses aplicadas e doses em dia na região Sul foi descrita segundo: estrato socioeconômico do setor censitário de residência (A, B, C e D), escolaridade materna (até oito anos de estudo, nove a 12 anos de estudo, 13 a 15 anos de estudo, 16 ou mais), idade materna (< 20 anos, 20 a 34 anos, 35 anos e mais), raça/cor da pele da mãe (branca, preta, parda, amarela, indígena), presença de companheiro (sim, não), número de filhos tidos (1, 2, 3+), uso de serviço privado em algum momento para vacinação (sim, não) e frequência a berçário/creche (sim, não). Os dados usados na classificação dos setores censitários de residência foram: renda média dos responsáveis pelo domicílio, proporção de responsáveis alfabetizados e proporção de responsáveis com renda maior ou igual a 20 salários mínimos, sendo A o estrato com melhor condição socieconômica e D o estrato com a pior.^1^

### Fontes de dados

Foi utilizado o banco de dados da região Sul do INCV 2020 já anonimizado, acessado no período de setembro a dezembro de 2023. Os dados do INCV 2020 foram coletados por meio de dispositivos eletrônicos em domicílio, segundo endereços do Sinasc e por busca ativa nos conglomerados sorteados, entre setembro de 2020 e março de 2022. Para identificação das doses aplicadas, foi fotografada cada carteira de vacinas e posteriormente digitados os dados. Informações socioeconômicas, comportamentais e sobre hesitação vacinal foram coletadas por meio da aplicação de questionário estruturado. Mais detalhes sobre o INCV 2020 estão descritos em publicações prévias.^1,13^

### Métodos estatísticos

Coberturas vacinais, proporções de atraso e intervalos de confiança foram calculados no programa Stata®, versão 17, utilizando-se o módulo *survey analysis*. Visto que a amostra do inquérito foi estratificada e conglomerada por setor censitário, considerando-se o estrato socioeconômico da área de residência, foi realizada a análise ponderada com pesos amostrais para cada um dos domicílios entrevistados, permitindo também evitar possíveis vieses decorrentes de perdas amostrais.^1^

Para identificar diferenças entre as coberturas, utilizou-se os intervalos de confiança de 95%.

### Considerações éticas

O inquérito foi aprovado pelos Comitês de Ética em Pesquisa com Seres Humanos do Instituto de Saúde Coletiva da Universidade Federal da Bahia, sob parecer número 3.366.818, em 4 de junho de 2019, com Certificado de Apresentação de Apreciação Ética (CAAE) 4306919.5.0000.5030; e da Irmandade da Santa Casa de São Paulo, sob parecer número 4.380.019, em 4 de novembro de 2020, com CAAE 39412020.0.0000.5479. Na coleta dos dados primários, foi utilizado o termo de consentimento livre e esclarecido dos responsáveis pelos lactentes. 

## RESULTADOS

A amostra final para a região Sul foi composta por 4.681 nascidos em 2017 e 2018, sendo 1.383 nascidos em Porto Alegre (RS), 1.192 em Curitiba (PR), 739 em Florianópolis (SC), 460 em Joinville (SC), 455 em Londrina (PR) e 452 em Rio Grande (RS). A perda amostral foi de 14% e ocorreu exclusivamente nos municípios de Curitiba (34%) e Florianópolis (18%), e foi mais frequente no estrato A.

A distribuição amostral segundo o perfil socioeconômico é apresentada na Tabela 1. Os participantes são em sua maioria dos estratos C e D. Quanto ao perfil das mães, foram observadas maiores proporções de escolaridade acima de 16 anos de estudo, idade acima de 35 anos, da cor branca, que possuem companheiro e com um único filho. Na amostra estudada, 36,9% dos lactentes utilizaram serviço privado de vacinação para alguma vacina.

**Tabela 1:** Perfil da amostra e cobertura vacinal completa aos 24 meses de doses aplicadas e doses em dia, segundo características socioeconômicas, em lactentes nascidos em municípios da região Sul do Brasil, 2020 a 2022 (n = 4.681)

Variáveis	Categorias	Amostra^a^		Cobertura vacinal (%)			
		**n**	**%**	**Doses aplicadas (IC**_95_ **%** **)**		**Doses em dia (IC**_95_ **%** **)**	
**Estrato socioeconômico da área de residência**	A	893	19,1	60,6	(53,6;67,1)	2,9	(1,6;5,5)
	B	1042	22,3	66,5	(55,6;75,9)	4,8	(1,8;12,1)
	C	1373	29,3	66,7	(59,1;73,5)	2,1	(1,3;3,4)
	D	1373	29,3	70,9	(65,1;76,2)	5,0	(2,9;8,3)
**Escolaridade materna (anos)**	≤ 12	898	19,5	61,9	(54,3;69,1)	2,5	(0,1;6,6)
	13 a 15	1.534	33,3	71,5	(66,0;76,4)	4,5	(2,6;7,8)
	16 ou mais	2.170	47,2	68,5	(61,6;74,6)	4,1	(2,1;7,9)
**Idade materna**	< 20 anos	59	1,3	32,9	(8,7;71,8)	0,0	-
	20 a 34 anos	2.251	48,2	68,5	(63,9;72,8)	3,1	(2,0;4,9)
	≥ 35 anos	2.358	50,5	69,9	(64,7;74,6)	5,1	(2,8;9,2)
**Raça/cor da pele da mãe**	Branca	3.603	78,4	70,3	(66,1;74,2)	4,1	(2,7;6,3)
	Preta	403	8,8	64,3	(54,3;73,2)	5,7	(1,5;9,4)
	Parda	552	12,0	60,1	(46,9;71,9)	1,8	(0,9;3,6)
	Amarela	35	0,8	70,8	(44,4;88,1)	0,3	(0,1;2,0)
	Indígena	2	0,0	38,0	(3,7;90,8)	0,0	-
**Mãe com companheiro**	Sim	3.740	81,5	70,3	(66,1;74,3)	3,2	(2,3;4,5)
	Não	847	18,5	61,0	(53,6;67,9)	6,6	(2,6;16,0)
**Número de filhos**	1	1.925	41,1	69,9	(63,7;75,5)	5,7	(3,4;9,5)
	2	1.846	39,4	65,8	(59,9;71,3)	3,5	(1,8;7,0)
	3+	911	19,5	68,7	(62,3;74,5)	1,2	(0,6;2,5)
**Uso do serviço privado** ^b^	Sim	1.719	36,9	67,8	(59,7;75,0)	4,5	(2,2;9,2)
	Não	2.935	63,1	68,1	(63,6;72,3)	3,6	(2,4;5,5)
**Frequenta berçário/creche**	Sim	2.764	59,1	68,8	(63,7;73,5)	4,7	(2,8;7,9)
	Não	1.912	40,9	67,1	(62,0;71,9)	2,8	(1,7;4,7)

a) Não foram apresentadas categorias com valores ignorados ou não informados; b) Para alguma vacina.

A cobertura das doses aplicadas e doses em dia para o conjunto de municípios participantes do INCV 2020, na região Sul do Brasil, é apresentada na Figura 1. A cobertura vacinal completa aos 24 para doses aplicadas foi de 68,0% (IC_95_% 63,9;71,8%) e para doses em dia foi de 3,9% (IC_95_% 2,7%;5,7%). Os valores de cobertura básica completa e cobertura completa aos 12 e 24 são similares quanto às doses aplicadas, sendo iguais a 79,6% (IC_95_% 76,1;83,2%) e 75,1% (IC_95_% 72,0;78,4%), respectivamente. Para as coberturas em dia, há maior proporção de doses aplicadas até os 12 meses (22,7%; IC_95_% 19,5;26,0%), quando comparada às vacinas indicadas de 12 e 24 meses (6,8%; IC_95_% 5,3;8,6%). Não foram observadas diferenças na cobertura completa para doses aplicadas e em dia segundo as variáveis analisadas (Tabela 1), exceto na comparação entre um filho e três filhos ou mais para doses em dia.

**Figura 1:** Cobertura vacinal de doses aplicadas e aplicadas em dia segundo esquema vacinal básico (aos 12 meses), de 12 a 24 meses e aos 24 meses, de lactentes nascidos em municípios da região Sul do Brasil, 2020 a 2022

Ao se analisar a cobertura de doses aplicadas segundo as vacinas (Figura 2), verifica-se que a maior parte das vacinas alcançou ou superou a cobertura de 90%. Apresentaram coberturas inferiores a 90% as vacinas: ROTA 2ª dose (87%), VIP 3ª dose (89%), MenC 1º reforço (87%), SCR 2ª dose (87%) e DTP 1º reforço (88%). Ao se analisarem as doses aplicadas em dia, observa-se que apenas duas vacinas alcançaram cobertura igual ou superior a 90% (HB e VPC10 1ª dose) e que as coberturas vão decrescendo conforme aumenta a idade de indicação da vacina, ficando abaixo de 50% para a MenC 1º reforço, SCR 2ª dose, VOP 1º reforço, DTP1º reforço e varicela 1ª dose. 

**Figura 2:** Cobertura vacinal para doses aplicadas e doses aplicadas em dia, para vacinas indicadas até os 24 meses, segundo doses, lactentes nascidos em municípios da região Sul do Brasil, 2020 a 2022

Com relação ao tempo de atraso (Figura 3), verifica-se a elevação do número de doses em atraso com o incremento da idade de indicação da vacina, bem como nas doses subsequentes dos esquemas com mais de uma dose e/ou reforço. A mesma situação ocorre na proporção de doses aplicadas com mais de três meses de atraso, alcançando maiores proporções nos primeiros reforços da DTP e VOP. A vacina HB é exceção, pois 56% das doses foram aplicadas com seis meses ou mais de atraso, ressaltando-se, contudo, que apenas 110 lactentes, atrasaram na aplicação dessa vacina.

**Figura 3:** Tempo de atraso das vacinas aplicadas em lactentes nascidos em municípios da região Sul do Brasil, 2020 a 2022

## DISCUSSÃO

A principal contribuição deste estudo é trazer informações recentes sobre a ocorrência do atraso vacinal em lactentes no cenário brasileiro. A cobertura de doses aplicadas e em dia na região Sul, para lactentes nascidos antes da pandemia de covid-19, identificou coberturas de doses aplicadas inferiores a 90% para cinco vacinas, com resultados preocupantes para doses em dia, chegando a coberturas inferiores a 50% para cinco das vacinas.

A cobertura completa de doses aplicadas, considerando-se todas as vacinas indicadas até os 24 meses, de aproximadamente 70%, embora mais elevada que a encontrada no país no mesmo inquérito (59,9%; IC_95_% 58,3;61,5),^1^ mostra o desafio para o alcance das metas de coberturas vacinais. Situação mais preocupante reside no fato de que apenas um em cada cinco lactentes recebeu as vacinas em dia até os 12 meses e menos de 10% cumpriu o calendário vacinal de rotina em dia até os 24 meses. Estudos nacionais e internacionais demonstram queda nas coberturas^1,8,15-19^ e ressaltam a preocupação quanto à maior vulnerabilidade na ocorrência de doenças imunopreveníveis.^14^ Ao avaliar cada vacina componente do esquema vacinal, fica evidente a queda progressiva nas coberturas de doses aplicadas, com menores coberturas para os imunobiológicos nas doses subsequentes (D2 e D3) e nas doses de reforço. Estudos em outros estados brasileiros que avaliaram imunobiológicos que possuem segundas doses e/ou doses de reforço mostraram maiores quedas na cobertura das doses subsequentes e/ou de reforço.^17,18^

Para a vacinação em dia, as coberturas são mais baixas, especialmente nas doses subsequentes, reforços e doses aplicadas acima de um ano. Pesquisa realizada em município do interior de São Paulo com nascidos em 2012 já apontava grande proporção de atraso vacinal para vacinas indicadas no segundo ano de vida, com 28,9% de crianças vacinadas oportunamente na segunda dose da SCR e nos reforços da DTP, VOP e pneumocócica.^19^ Estudo que avaliou a prevalência do atraso nas três doses da vacina tetravalente (DTP+ *haemophilus influenzae* tipo B) em crianças de 12 a 23 meses de idade, no Brasil, com dados da Pesquisa Nacional de Saúde de 2013, apontou atraso de 14,8% na primeira, 28,8% na segunda e 45,4% na terceira dose.^11^ Outros países também apresentam incremento de atraso vacinal segundo as doses.^20-24^ Tais achados indicam a necessidade de reforço de orientações quanto à completude do esquema vacinal, incluindo reforços no momento oportuno, principalmente no segundo ano de vida, quando a frequência de atraso e abandono é maior.

Embora as coberturas em dia segundo o perfil socioeconômico apontem proporções mais elevadas entre os lactentes de mães com maior escolaridade, menor número de filhos, com companheiro e residentes no estrato D, e que frequentam creche/berçário, não foram identificadas diferenças estatisticamente significativas quanto à cobertura de doses aplicadas e em dia, contrariando estudos anteriores que demonstram associação com fatores socioeconômicos, como escolaridade e renda. Enquanto estudos realizados nas regiões Norte e Nordeste do país apontam piores coberturas em estratos de menor renda e escolaridade,^15,16^ em São Paulo foi identificado aumento da cobertura conforme decréscimo do nível socioeconômico.^25^ Avaliação de atraso vacinal para a vacina tetravalente em 2013, no Brasil, apontou maior proporção de atraso em crianças pardas, pertencentes a famílias mais pobres, moradores da zona rural e da Região Norte.^11^ Dados de 2008 a 2018 de crianças vacinadas na Inglaterra apontaram relação inversa entre vacinação oportuna e nível socioeconômico, além de diferenças segundo as regiões do país.^20^

Ao avaliar o tempo de atraso, observaram-se diferenças segundo doses, sendo menor para as primeiras e segundas doses e maior para as terceiras doses, os reforços e as doses aplicadas a partir de 12 meses. Para os reforços da DTP e VOP, o atraso foi superior a 60%, e um em cada quatro lactentes que teve a vacinação atrasada recebeu a vacina num intervalo maior que seis meses após a data recomendada para vacinação. O atraso na vacinação acarreta o prolongamento da exposição do lactente a doenças imunopreveníveis, mais incidentes na infância, e ainda aumenta o risco de não se completar o calendário vacinal de rotina.^13,19^ A exemplo disso, destaca-se a ocorrência de surtos de sarampo no mundo por conta de atrasos na vacinação, apesar da alta cobertura global.^19^ Estudo em hospital da Arábia Saudita, realizado em 2018, considerando as vacinas do calendário infantil do país, revelou que 59,1% das crianças atrasaram a vacinação em ao menos um mês.^26^ Em Montana, nos Estados Unidos, apenas 38,0% dos lactentes avaliados de 2015 a 2019 receberam todas as doses da vacina em dia.^27^ Kurosky et al.,^28^ ao avaliarem dados do inquérito nacional de 2012 nos Estados Unidos, apontaram, além da baixa vacinação em dia, atrasos prolongados de até sete meses. 

As altas proporções de atraso vacinal apontam para a necessidade de os serviços de vacinação, especialmente a atenção primária, reforçarem ações para garantir a vacinação em dia. A utilização de estratégias de lembrete de data de vacinas próximas é recomendada nos Estados Unidos pelo Grupo de Trabalho sobre Serviços Preventivos Comunitários para aumentar as taxas de imunização.^29^ Com o avanço das tecnologias de comunicação, pode-se utilizar o envio de mensagens por meio de *e-mail* e celular, além do reforço às orientações pelos profissionais da puericultura e das salas de vacinas para aumentar a confiança nas vacinas e quanto à importância de manter a vacina em dia.^27,29,30^ Além disso, ampliar a cobertura da Estratégia Saúde da Família, com equipes completas e atuação dos Agentes Comunitários de Saúde na busca ativa, são medidas importantes. Diminuir barreiras de acesso, sejam elas materiais, relacionadas às famílias, sejam as urbanas e aquelas vinculadas aos serviços de saúde, deve ser prioridade para os gestores municipais no Brasil.

Algumas limitações do presente estudo precisam ser destacadas. Observaram-se perdas amostrais, em especial nos municípios de Curitiba e Florianópolis, influenciadas também pelo momento de coleta dos dados, que coincidiu com o período de medidas de distanciamento social devido à pandemia de covid-19. Contudo, estratégias de expansão territorial para a coleta de dados e a utilização de pesos amostrais, considerando-se os grupos em que houve maior perda, minimizaram os efeitos dessa perda amostral. Destaca-se que os dados analisados são relacionados à região Sul, não podendo ser generalizados para o restante do país. Na amostra da região Sul, havia maior proporção de mães com escolaridade de 16 anos ou mais de estudo, idade acima 34 anos e da raça/cor da pele branca, características distintas do observado no perfil de entrevistados do INCV 2020 no país.^1^

Considerando-se que o presente estudo descreveu o panorama de seis cidades no sul do país, mostra-se importante a realização de novos estudos para se conhecer a realidade de outras regiões do país e do contexto nacional, não somente relativos à cobertura vacinal, mas também que avaliem o atraso vacinal e os fatores associados. 

O estudo ainda aponta que, além de garantir a vacinação, é de extrema importância seguir corretamente o calendário vacinal. Visto que as coberturas são avaliadas, em geral, ao final do primeiro e segundo anos de vida, vários meses após a idade recomendada para as vacinas, é fundamental que os sistemas de monitoramento de vacinação acompanhem se as vacinas estão sendo aplicadas nas idades preconizadas, com a adoção de estratégias que reforcem a vacinação de rotina para a prevenção do atraso vacinal, ação essencial da atenção primária. A orientação aos responsáveis sobre a necessidade de manter o calendário em dia e a adoção de estratégias de aviso de doses próximas devem ser reforçadas, não se concentrando os esforços somente em campanhas de busca de faltosos.
